# Targeting YAP‐p62 signaling axis suppresses the EGFR‐TKI‐resistant lung adenocarcinoma

**DOI:** 10.1002/cam4.3734

**Published:** 2021-01-23

**Authors:** Hee Sun Park, Da‐Hye Lee, Da Hyun Kang, Min‐Kyung Yeo, Goeun Bae, Dahye Lee, Geon Yoo, Ju‐Ock Kim, Eunyoung Moon, Yang Hoon Huh, Sang‐Hee Lee, Eun‐Kyeong Jo, Sang Yeon Cho, Jeong Eun Lee, Chaeuk Chung

**Affiliations:** ^1^ Division of Pulmonology Department of Internal Medicine College of Medicine Chungnam National University Daejeon Republic of Korea; ^2^ Division of Chemical and Biological metrology Korea Research Institute for Standards and Science Daejeon South Korea; ^3^ Department of Pathology College of Medicine Chungnam National University Daejeon Republic of Korea; ^4^ Korea Institute of Toxicology Daejeon Republic of Korea; ^5^ Electron Microscopy Research Center Korea Basic Science Institute (KBSI Cheongju‐si Republic of Korea; ^6^ Department of Microbiology Chungnam National University School of Medicine Daejeon Republic of Korea; ^7^ Infection Control Convergence Research Center Chungnam National University School of Medicine Daejeon Republic of Korea; ^8^ Department of Medical Science Chungnam National University School of Medicine Daejeon Republic of Korea; ^9^ Chungnam National University Schoolof Medicine Daejeon Republic of Korea

**Keywords:** autophagy, EGFR‐TKI, lung adenocarcinoma, p62, YAP

## Abstract

**Background:**

Despite the progress of advanced target therapeutic agents and immune checkpoint inhibitors, EGFR‐TKI resistance is still one of the biggest obstacles in treating lung cancer. Clinical studies with autophagy inhibitors are actively underway to overcome drug resistance.

**Methods:**

We used PC9, PC9/GR, and HCC827/GR cell lines to evaluate the activation of autophagy and EGFR‐TKI resistance. Chloroquine was applied as an autophagic blocker and verteporfin was utilized as a YAP inhibitor.

**Results:**

In this study, we tried to reveal the effect of autophagy adaptor p62 which is accumulated by autophagy inhibitor in EGFR‐TKI‐resistant lung adenocarcinoma. We identified that p62 has oncogenic functions that induce cell proliferation and invasion of EGFR‐TKI‐resistant lung adenocarcinoma. Interestingly, we found for the first time that YAP regulates p62 transcription through ERK, and YAP inhibition can suppress the expression of oncogenic p62. We also confirmed that the expressions of p62 and YAP have a positive correlation in EGFR‐mutant lung adenocarcinoma patients. To block cell survival via perturbing YAP‐p62 axis, we treated EGFR‐TKI‐resistant lung cancer cells with YAP inhibitor verteporfin. Remarkably, verteporfin effectively caused the death of EGFR‐TKI‐resistant lung cancer cells by decreasing the expressions of p62 with oncogenic function, YAP, and its target PD‐L1. So, the cumulative effect of oncogenic p62 should be considered when using autophagy inhibitors, especially drugs that act at the last stage of autophagy such as chloroquine and bafilomycin A1.

**Conclusion:**

Finally, we suggest that targeting YAP‐p62 signaling axis can be useful to suppress the EGFR‐TKI‐resistant lung cancer. Therefore, drug repurposing of verteporfin for lung cancer treatment may be valuable to consider because it can inhibit critical targets: p62, YAP, and PD‐L1 at the same time.

## Graphical abstract

The accumulated p62 by autophagy inhibitor, chloroquine has oncogenic functions in EGFR‐TKI resistant lung adenocarcinoma. Hippo effector YAP regulates p62 transcription through ERK, and YAP inhibition significantly suppresses the expression of p62. We suggest that targeting YAP‐p62 signaling axis can be useful to overcome the EGFR‐TKI resistance in lung adenocarcinoma.

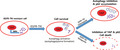

## 1 INTRODUCTION

Lung cancer treatment has markedly improved mainly due to the development of innovative‐targeted therapy and immunotherapy. But the effect of those novel therapies is also limited due to the drug resistance. Although third generation EGFR‐TKIs are effective for even EGFR‐TKI‐resistant cells with acquired T790M mutation, there are still few effective drugs for other resistant mechanisms.^1^ Cancer immunotherapy has recently gained a spot light for its long‐lasting response, but the overall response rate of immunotherapy is around 20%–30% due to primary resistance. There are also many cases of acquired resistance to immunotherapy, which can be driven by several mechanisms such as immunoediting and activation of oncologic signaling.^2^, ^3^ So, in order to overcome this drug resistance, numerous works are currently underway to identify novel targets, and autophagy is one of the most actively studied topics.

Autophagy is a physiologic process that can be activated to cope with cellular stresses such as serum starvation or increased metabolic demands.^4^, ^5^ Interestingly, autophagy is known to have opposed, context‐dependent functions in cancer. Before the establishment of a tumor, autophagy acts as a tumor suppressor by degrading the misfolded proteins. But in the established cancer cells, autophagy activation can function as a survival mechanism against chemotherapy and radiation therapy.^6^ Recent studies showed that autophagy inhibitors such as hydroxychloroquine (HCQ) and chloroquine were helpful to restore chemosensitivity by inhibiting the fusion between autophagosome and lysosome.^5^, ^7^ Clinical trials using autophagy‐targeting drugs either as monotherapy or in combination therapy are undergoing. Although some studies showed promising effects of these autophagy inhibitors in intractable patients, side effects such as retinopathy and gastrointestinal problems due to HCQ and chloroquine are issued, and the proper indications for these drugs are not yet to be determined.^6^, ^8^, ^9^, ^10^ Sequestosome 1 (p62/SQSTM 1) functions as an autophagy adaptor and is degraded like other autophagy substrates during the autophagic flux.^11^ Because p62 also has an oncogenic function in some contexts, maintaining an appropriate level of p62 is very important to prevent tumorigenesis and inhibit cancer progression.^11^, ^12^ So, in the normal cell, the expression of p62 is finely regulated by many important signalings including oxidative stress, JNK, ERK, and NRF2. In autophagic defective cells, accumulated p62 can cause genomic instability and tumorigenesis by interacting with oncogenic signalings.^11^, ^13^, ^14^

The Hippo pathway is basically important in the development and plays critical functions in a wide range of pathologic processes, including tumorigenesis, metastasis, and epithelial‐mesenchymal transition.^15^, ^16^ The Hippo effector YAP confers EGFR‐TKI resistance by many mechanisms including the upregulating AXL and ERK activation in lung cancer.^17^ And, more recent paper showed that YAP can directly regulate the transcription of PD‐L1; target of an immune checkpoint inhibitor.^18^ Verteporfin, an FDA‐approved drug that interrupts YAP/TEAD interaction, is used as a photosensitizer for macular degeneration.^19^ Some studies have revealed verteporfin has promising effects in overcoming the chemo‐resistance in certain contexts.^17^, ^20^, ^21^, ^22^

Nowadays, the interaction between YAP and autophagy is an intriguing topic in the biological and medical fields. Some studies demonstrated that YAP activates autophagy to promote cancer cell survival or resistance of cisplatin.^23^, ^24^ Inversely, a study showed that autophagy regulates the carcinogenesis and differentiation of liver cells by the degradation of YAP.^25^ But the mechanisms and effects of their interaction are very different depending on contexts, so the relationship between YAP and p62 still remains to be elucidated.

In this study, we identified that lung cancer cells with EGFR‐TKI resistance activate autophagy in response to EGFR‐TKI and emphasized the effect of oncogenic p62, which is accumulated by autophagy inhibitor chloroquine. Our results revealed for the first time that the relationship between autophagy adapter p62 and YAP in lung adenocarcinoma with laboratory experiments and clinical data. We also studied whether targeting YAP‐p62 signaling and YAP inhibitor verteporfin can be used to suppress EGFR‐TKI resistance in lung adenocarcinoma.

## 2 MATERIALS AND METHODS

### 2.1 Cell lines and reagents

The human lung cancer cell lines, PC9, PC9/GR, and HCC827/GR cells were cultured at 37°C in 5% CO2 in RPMI‐1640 media (WELGENE) containing 10% fetal bovine serum (FBS) (WELGENE). The PC9, PC9/GR, and HCC827/GR cell lines were kindly provided by Dr. JC Lee of the Department of Oncology, University of Ulsan, Asan medical center, Republic of Korea. EGFR‐Tyrosine kinase inhibitor (Gefitinib) was purchased from Tocris (Iressa, 184475‐35‐2). Chloroquine (C6628), Bafilomycin A1 (B1793), Verteporfin (SML0534), and PD98059 (P215) were purchased from Sigma‐Aldrich.

### 2.2 Transient transfection

Prof. DS Lim (KAIST) provided pDKflag‐YAPWT, pDKflag‐YAP2SA, and control vector plasmids. Prof. EK Jo (CNU) provided mRFP‐GFP tandem fluorescent‐tagged LC3 construct. The transfections of different DNA constructs were performed using Lipofectamine 2000 (Invitrogen) as said by the manufacturer's instructions. Further assays were conducted after 48 h incubation of transiently transfected cells.

### 2.3 Immunofluorescence

We fixed cultured cells with 4% paraformaldehyde at room temperature, permeabilized with 0.1% Triton X‐100 in PBS, and blocked with 3% FBS in PBS. Following overnight incubation at 4°C with primary antibodies (LC3; PM036, MBL), and incubation in the dark with Alexa Fluor 594 Fluor dye‐labeled secondary antibodies, immunofluorescence was detected using a fluorescence microscope (Leica).

### 2.4 Autophagic flux assay

For autophagic flux assay, we transfected tandem fluorescent‐tagged LC3 (mRFP‐EGFP‐LC3) construct into PC9/GR cells and PC9 cells. We washed the cells twice in ice‐cold PBS, fixed, mounted with Histological Mounting medium and then, observed with a fluorescence microscope (OLYMPUS).

### 2.5 Luciferase reporter assay

The PC9 cells seeded in 100 mm plate were transfected with pDKflag‐YAPWT and pGL3‐p62luciferase reporter (given by TI Jeon, Chonnam National University) using Lipofectamine 2000 reagent (Invitrogen) for 48 h. Cells were harvested and assayed using the Luciferase assay system (Promega Corp) and luminescent signaling was detected using a Lumat LB9507 Luminometer (Berthold Detection System) as said by the instruction of manufacturer. We determined protein concentrations of the cell lysate by the Bradford method. We normalized firefly luciferase activity to cell protein content. We performed all the assays in triplicate. We expressed the means of relative luciferase units in the fold with respect to basal expression of the pGL3 promoter vector (given by TI Jeon, Chonnam National University).

### 2.6 Cell viability assay and Wound healing assay

We seeded the cells into 6‐well plates to 80%–90% confluence and scratched the cell monolayer in a straight line using a 200 μl pipette tip. We took the images at a 0 and 18 h after the scratch to calculate the cell migration rate. We counted cell viability using the CCK‐8 assay kit (Dojindo Laboratories) thrice in triplicate.

### 2.7 Invasion assays and Transwell migration

We performed migration and invasion assays using Transwell membrane (0.8 μm pore‐size cell culture insert, FALCON). For cell migration assay, we coated a non‐coated Transwell membrane and for cell invasion assay Transwell membrane with 2 mg/ml Matrigel (354234, Corning). For migration assays and invasion assay, we seeded the cells in serum‐free medium onto the upper chamber and added 750 μl growth medium with 10% FBS to the lower chamber. After incubation for 18 h, we removed the cells that did not migrate through or invade the pores with a cotton swab. We fixed the migration and invasion cells, which adhered to the lower surface with 4% paraformaldehyde and stained with 0.1% crystal violet. We counted the cells using a light microscope in three randomly selected fields. We performed the experiments thrice in triplicate.

### 2.8 Correlation and survival analysis

Cut off Finder (http://molpath.charite.de/cutoff) was used to determine the cutoff values for LUAD mRNA expression. The determination of optimal cutoff point in Cut off Finder was based on the overall survival rate for significance on the basis of the log‐rank test for the outcome of patients (http://molpath.charite.de/cutoff/assign.jsp). We performed the Kaplan–Meier method to estimate the cumulative event (death) rate from the date of operation until death used as the outcome variable. Survival curves stratified by high and low expression groups on YAP1genes were compared using the log‐rank test. A *p*‐value < 0.05 was considered to indicate a statistically significant difference.

### 2.9 Statistical analysis

We used GraphPad Prism version 5.0 software (GraphPad) for analysis differences between two groups (Student's *t*‐test) and for performing nonlinear regression analysis. A *p*‐value < 0.05 was considered to be statistically significant.

## 3 RESULTS

### 3.1 EGFR‐TKI treatment activates autophagy in both PC9 and PC9/GR cells

**FIGURE 1:**
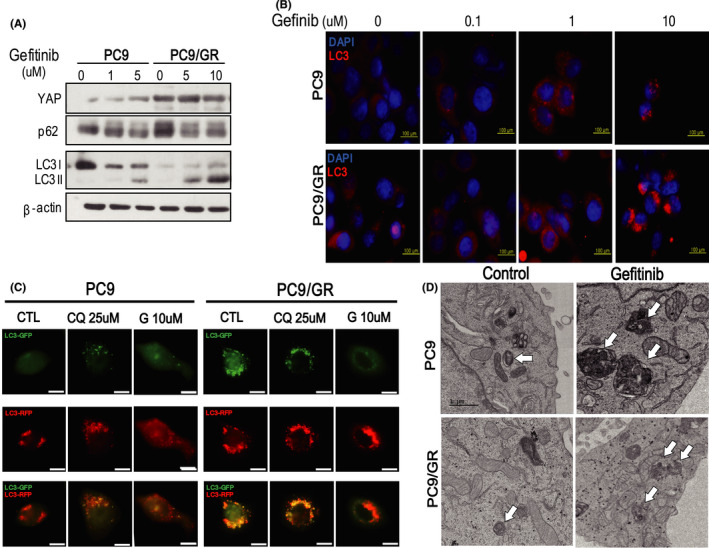
EGFR‐TKI treatment activates autophagy in PC9 and PC9/GR cells. (A) Western blot analysis of lysates derived from PC9 and PC9/GR cells treated for 6 h with increasing concentration of gefitinib. (B) Analysis of LC 3 aggregation in PC9 and PC9/GR cells by fluorescence microscopy. The LC3 puncta were determined after treatment of 0, 0.1, 1, and 10 μM gefitinib. At a high dose of gefitinib, most of PC9 cells died (Scale bar, 200 µm). (C) Immunofluorescent staining of PC9 and PC9/GR cells transfected with mRFP‐GFP tandem fluorescent‐tagged LC3 construct. Chloroquine blocked the fusion of autophagosome and lysosome, causing GFP accumulation, however, gefitinib caused a significant decrease of GFP in both cell lines, indicating the activation of autophagic flux. (D)TEM images of PC9 and PC9/GR cells showed that gefitinib treatment increased cytoplasmic organelles with a double lumen in both cell lines, suggesting activation of autophagy. Representative images are shown, and the arrows indicate autophagosomes (scale bar, 1 µm)

To find out the alterations of autophagy in PC9 and PC9/GR after treatment of EGFR‐TKI, we first checked the expression levels of LC3‐I/LC3‐II and p62 with western blot and immunofluorescent assay. We discovered that ratios of LC3‐II to LC3‐I were markedly increased and the expressions of p62 were significantly decreased in both PC9 and PC9/GR after EGFR‐TKI treatment (Figure 1A; Figure S1A). The immunofluorescent study showed both PC9 and PC9/GR had much more LC3 puncta after EGFR‐TKI treatment (Figure 1B). These data suggest that EGFR‐TKI induces autophagic flux in EGFR‐TKI sensitive PC9 and even EGFR‐TKI‐resistant PC9/GR cells. To confirm the autophagic flux, mRFP‐GFP tandem fluorescent‐tagged LC3 construct was transfected into PC9 and PC9/GR cells. It can be used to visualize the autophagic flux because the fluorescence of GFP but not of mRFP is quenched within acidic autolysosomes.^26^ As expected, chloroquine which inhibited lysosomal acidification and blocking autophagosome‐lysosome fusion hindered quenching of GFP,^27^, ^28^ showing inhibition of autophagic flux. Moreover, EGFR‐TKI treatment caused significantly decreased expression of GFP of both cell lines, indicating the activation of autophagic flux (Figure 1C). Consistently, electron microscopy (EM) data confirmed that there were a few autophagosomes in PC9 at the baseline, but after EGFR‐TKI treatment there were increased number of autophagosomes. Interestingly, PC9/GR also has much more autophagosomes after EGFR‐TKI treatment. Taken together, we found that EGFR‐TKI treatment activates autophagy in both PC9 cells and PC9/GR cells (Figure 1D), suggesting activation of autophagy might be one of the survival mechanisms against EGFR‐TKI.

### 3.2 **Autophagy blocker chloroquine causes the accumulation of oncogenic p62 which promotes cell survival and invasion of PC9/GR cells**.

**FIGURE 2:**
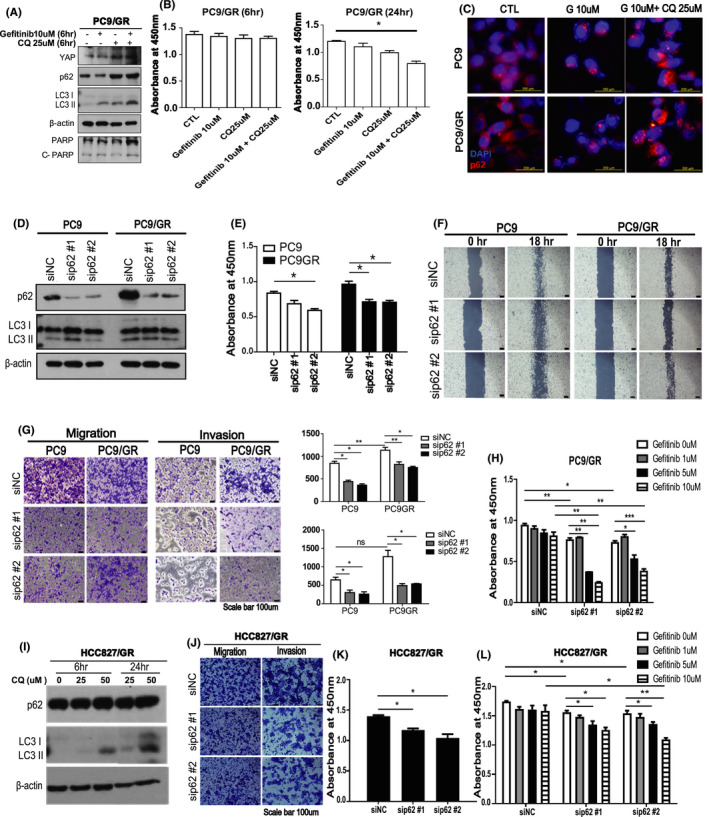
Autophagy inhibitor chloroquine causes the accumulation of oncogenic p62 which promotes cell proliferation, migration, and invasion. (A) Western blot analysis of lysates derived from PC9/GR cells treated with gefitinib (10 mM) or chloroquine (25 μM) for 6 h. (B) The left and right panel are the results of CCK assay of PC9/GR after treatment of gefitinib (10 mM) or chloroquine (25 μM) for 6 and 24 h, respectively. Data are means ± SD; *p < 0.05, Student's *t*‐test. (C) The expressions of p62 of PC9 and PC9/GR cells were determined by immunofluorescent staining after treatment of gefitinib (10 mM) or a combination of gefitinib and chloroquine (25 μM) for 6 h, showing chloroquine caused significant p62 accumulation in PC9 and PC9/GR. (Scale bar, 200 µm) (D) Western blot of lysates derived from PC9 and PC9/GR cells after the treatment of siRNA p62. (E) The CCK assay assessing cell viabilities of control and p62 knock downed cells. (F) Wound healing assay. We transfected the PC9 and PC9/GR cells with the vector or p62 siRNA. Movement of cells into wounds is shown at 0 and 18 h post‐scratching. (Scale bar: 200 µm) (G) Representative images of the migration and invasion assay. We used a non‐coated transwell membrane for cell migration assay and transwell membrane coated with 2 mg/ml Matrigel was used for cell invasion assay. The PC9 and PC9/GR cell lines were transfected with the vector or p62 siRNA. After incubation 18 h, cells that did not migrate through or invade the pores were removed with a cotton swab. Cells were counted for the corresponding assays of at least three random microscope fields. The right panel shows quantification of the results. (H) We assed cell viability by CCK assay after a combination treatment of gefitinib with control or p62 siRNA. The Addition of p62 knock down significantly decreased the viability in PC9/GR cell. (I) Western blot of p62, LC3‐I/II, and β‐actin after treatment of chloroquine in HCC827/GR cell. (J) Representative images of the migration and invasion assay. We used a non‐coated transwell membrane for cell migration assay and transwell membrane coated with 2 mg/ml Matrigel was used for cell invasion assay. The HCC827/GR cell lines were transfected with the vector or p62 siRNA. (K) CCK assay after treatment of p62 siRNA in HCC827/GR cells. (L) CCK assay after treatment of p62 siRNA with control or several doses of gefitinib in HCC827/GR cells. Data are means ± SD; *p < 0.05; **p < 0.01; ***p < 0.005

As previously mentioned, chloroquine can inhibit autophagosome–lysosome fusion and it is most commonly used as an autophagy blocker. To check the effect of combined treatment of autophagy blocker and EGFR‐TKI, we treated gefitinib and chloroquine together in PC9/GR cells. Twenty‐four h combined treatment of gefitinib and chloroquine significantly decreased cell proliferation of PC9/GR, while 6 h combined treatment did not make a significant difference (Figure 2A,B; Figure S2A). The high dose of gefitinib and chloroquine more effectively decreased the PC9/GR cell proliferation than the low dose of the drugs (Figure S2C,D).

We found that there was a substantial accumulation of p62 in chloroquine‐treated cells (Figure 2A,C). As autophagic adaptor p62 can induce genomic instability and tumor progression in some contexts,^12^ we studied the function of p62 in EGFR‐TKI‐resistant PC9/GR cells. To confirm the oncogenic role of p62, we knocked down p62 using siRNA in PC9 and PC9/GR. The results showed that knock down of p62 significantly decreased the cell proliferation and the capacity of wound healing in the PC9 and PC9/GR cells (Figure 2D–F; Figure S2B). Inhibitory effect on PC9 and PC9/GR cells by high concentration of p62 siRNA was significantly stronger than low concentration of p62 siRNA (Figure S2E).

Further experiments demonstrated that p62 knockdown meaningfully decreased the migration and invasion of both cell lines (Figure 2G). Although PC9/GR cell is characterized by resistance to high doses of gefitinib, co‐treatment of gefitinib with p62 siRNA considerably overcame the resistance of EGFR‐TKI (Figure 2H). To validate these results, we used another gefitinib resistance cell lines, HCC827/GR cells. Consistent with PC9 and PC9/GR, chloroquine treatment caused the accumulation of p62 in HCC827/GR cells (Figure 2I). Knock down of p62 significantly decreased the cell proliferation and suppressed the migration and invasion in HCC827/GR cells (Figure 2J–L; Figure S2F). These data suggest that p62 has oncogenic functions in EGFR‐TKI‐resistant cells and blocking p62 might be useful for suppressing the EGFR‐TKI resistance.

### 3.3 YAP regulates the expression of p62 via ERK in PC9 and PC9/GR cell

**FIGURE 3:**
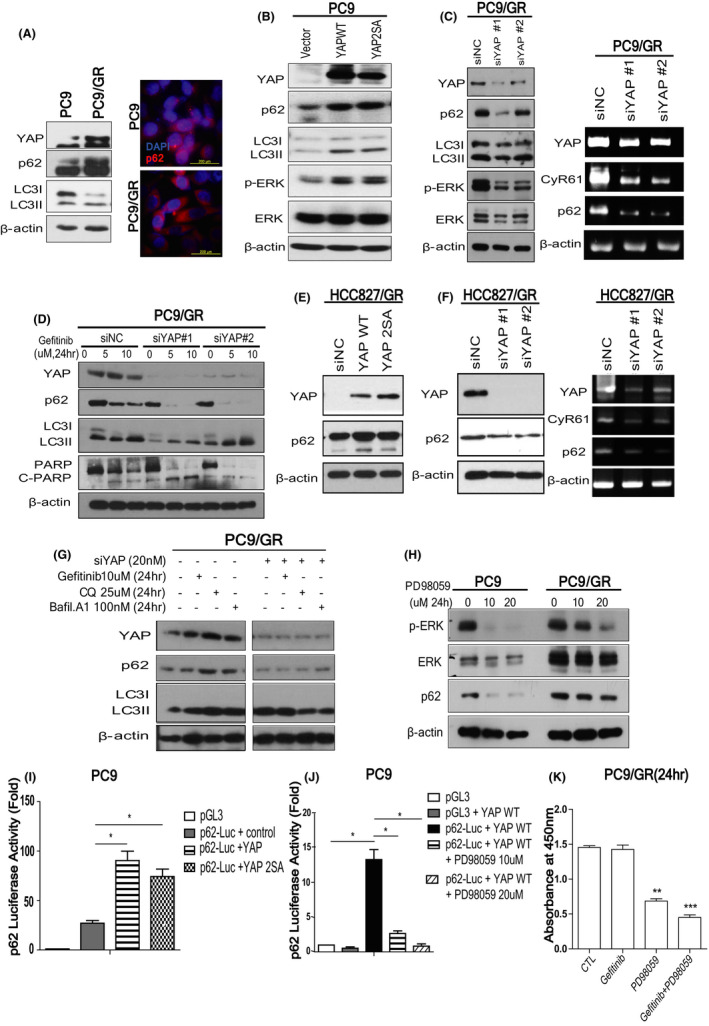
YAP regulates the transcription of p62 via ERK in PC9 and PC9/GR cell. (A) The left panel is the western analysis of YAP and p62, LC3‐I/II in PC9 and PC9/GR. The right panel is the immunofluorescent staining of p62 in PC9 and PC9/GR. (B) Western blot analysis of YAP, p62, LC3‐I/II, ERK, and phospho‐ERK in PC9 control and YAP‐overexpressing PC9 cells (wild‐type YAP and YAP 2SA, respectively). (C) The left panel is the western blot analysis of YAP, p62, LC3‐I/II, ERK, and phospho‐ERK in PC9/GR control and YAP knock downed PC9/GR. The right panel shows the mRNA expressions of YAP, Cyr61, p62, and β‐action determined by RT‐PCR. (D) The western blot of YAP, p62, LC3‐I/II, PARP, and C‐PARP after treatment of gefitinib with siYAP or control. (E) Western blot of p62, YAP, and β‐actin after overexpression of wild‐type YAP or constitutively active YAP, YAP 2SA in HCC827/GR cells. (F) The left panel is western blot of p62, YAP, and β‐actin after knock down of YAP using YAP siRNA #1 and #2 in HCC827/GR cells. The right panel is the mRNA expressions of p62, CyR61, and β‐actin in HCC827/GR cell after knock down of YAP using YAP siRNA #1 and #2. (G) The western blot of YAP, p62, and LC3‐I/II after treatment of gefitinib, CQ, bafilomycin A1, or siYAP. (H) Western blot of ERK, phospho‐ERK, and p62 after treatment of ERK inhibitor, PD98059 for 24 h (0, 10, and 20 μM, respectively). (I) Luciferase assay using ofpGL3‐p62vector and pDKflag‐YAP (wild‐type YAP and YAP 2SA, respectively). (J) Luciferase assay using pGL3‐p62vector after YAP overexpression with or without ERK inhibitor, PD98059. Data (mean ± standard error) are from a representative experiment conducted in triplicates and are expressed as fold changes relative to the cells harboring control plasmid. (K) CCK assay of PC9/GR cells after treatment of gefitinib or ERK inhibitor, PD98059. *p < 0.05, ***p < 0.001 (Student's *t*‐test)

In the previous study, we demonstrated that Hippo effector YAP is deeply tangled in EGFR‐TKI resistance and PD‐L1 transcription.^17^, ^20^ Recent published works demonstrated that YAP, PD‐L1, and autophagy are closely related to each other.^25^, ^29^, ^30^, ^31^, ^32^ But the relationship between YAP and autophagic adaptor p62 has not been revealed. Interestingly, we found that both YAP and p62 expressions of PC9/GR cells were much higher than those of PC9 cells (Figure 3A; Figure S3A). To check the direct regulation between them, we overexpressed YAP with wild‐type YAP and YAP 2SA and knock downed YAP using siRNA in PC9 and PC9/GR cells. First, YAP overexpression significantly augmented not only protein expression, but also mRNA level of p62 in PC9 cells (Figure 3B; Figure S3B). Knock down of YAP also significantly decreased p62 expression of both protein and mRNA in PC9/GR cells (Figure 3C; Figure S3C). In addition to p62, the expression of phospho‐ERK was affected by YAP (Figure 3B,C). Then, we checked the effect of the combination of siYAP and gefitinib on p62. Consistent with the previous data (Figure 1A), gefitinib decreased p62 expression, and the combined treatment of siYAP and gefitinib almost eliminated the remaining p62 expression (Figure 3D; Figure S3D). Consistent with PC9 and PC9/GR, this regulation of YAP on p62 was also confirmed in HCC827/GR cells (Figure 3E,F; Figure S3E–G).

Finally, to exclude the degradation of p62 by autophagic flux, we treated siYAP with autophagic inhibitors including chloroquine and bafilomycin A1. Bafilomycin A1 inhibits autophagy by targeting V‐ATPase in the lysosome.^33^ As expected, autophagy inhibitors caused the accumulation of p62 in PC9/GR by blocking autophagic flux. Notably, the addition of siYAP significantly diminished the expression of p62 even in a combination of autophagic inhibitors, suggesting that knock down of YAP decreased the expression of p62 independently of autophagic flux (Figure 3G). Overall, these data suggest co‐transcriptional factor YAP regulates the transcription of p62. So, we next checked whether YAP/TEAD complex could bind on the p62 promoter or enhancer, but there was no site to which the complex could bind (Figure S5A–C). So, we concluded that YAP regulates the transcription of p62 indirectly. p62 is known to be finely regulated by autophagic degradation and many signals including JNK/c‐Jun pathway, NRF2, oxidative stress, and RAS/MAPK pathway.^34^ And, YAP is also known to activate ERK signaling in lung adenocarcinoma.^17^ Our results showed that YAP knockdown significantly deceased the phospho‐ERK and p62 at the same time, whereas YAP overexpression increased the phospho‐ERK and p62 together (Figure 3B,C). So, to confirm whether ERK is a mediator between YAP and p62 interaction, we treated ERK inhibitor, PD98059 in both PC9 and PC9/GR cells. Treatment with 10 μM ERK inhibitor markedly lessened the expression of p62 in PC9 cells, while 20 μM ERK inhibitor could inhibit ERK activation and decrease the expression of p62 in PC9 and PC9/GR cells (Figure 3H). In addition, YAP expression increased the endogenous p62 expression in PC9/GR cells, while ERK inhibitor decreased the p62 expression which was increased by YAP (Figure S3H). To directly address the hypothesis that p62 transcriptional activity is regulated by YAP via ERK, we performed luciferase reporter assay with pGL3‐p62 promoter. The results indicated that YAP overexpression significantly increased the transcription of p62 and inversely, ERK inhibitor almost depleted the transcription of p62 which were induced by YAP (Figure 3I,J; Figure S3I). Furthermore, to confirm the effect of ERK inhibitor on suppressing the gefitinib resistance, we treated gefitinib and ERK inhibitor to PC9 and PC9/GR cells. The combination treatment of ERK inhibitor and gefitinib significantly recovered the sensitivity of gefitinib in PC9/GR (Figure 3K). Overall, these results suggested that YAP stimulates the transcription of p62 throughout the activation of ERK and targeting YAP‐ERK‐p62 signaling may be feasible strategy to suppress the EGFR‐TKI resistance.

### 3.4 YAP inhibition reduces expression of YAP, PD‐L1, and p62 at the same time, significantly overcoming EGFR‐TKI resistance in lung cancer

**FIGURE 4:**
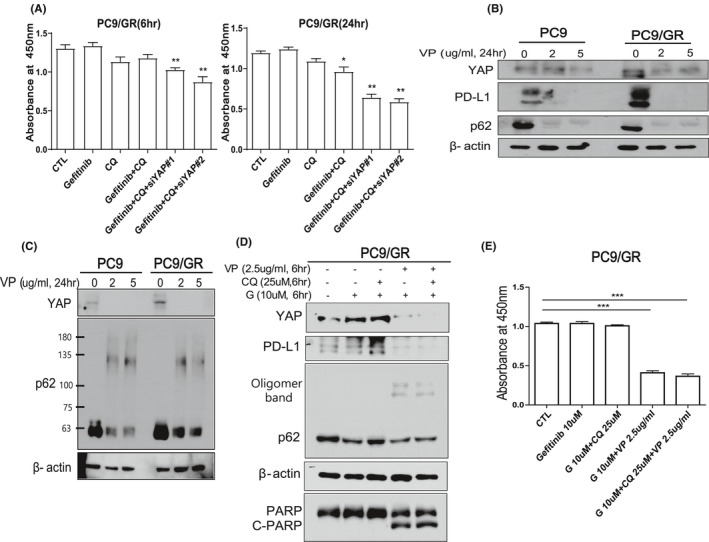
YAP inhibitor, Verteporfin (VP) suppresses the cell proliferation of EGFR‐TKI‐resistant PC9/GR. (A) CCK assay of PC9/GR cells after treatment of 5 μM gefitinib or 25 μM CQ with YAP siRNA#1 or siRNA #2. Left and right panels are the results of 6 and 24 h treatments, respectively. (B) Western blot analysis of YAP and p62 in PC9 and PC9/GR cells after treatment of VP for 24 h (0, 2, and 5 μg/ml, respectively). VP significantly decreased the expression of YAP, its target PD‐L1, and p62. (C) Western blot analysis of YAP and p62 with partial p62 oligomerization in PC9/GR cells after treatment of VP for 24 h (0, 2, and 5 µg/ml, respectively). (D) Western blot analysis of YAP, PD‐L1, p62, PARP, and C‐PARP in PC9/GR cells after combination treatment of gefitinib (10 μM) with VP (2.5 μg/ml) or chloroquine(25 μM) for 6 h. (E) Cell viability was assessed by CCK assay in PC9/GR cell after combination treatment of gefitinib (10 μM) with VP (2.5 μg/ml) or chloroquine (25 μM) for 6 h. ***p < 0.005 (Student's *t*‐test)

Based on the result that YAP regulates the expression of oncogenic p62 expression, we added the YAP knock down to the combination treatment of chloroquine and gefitinib in PC9/GR cells. The combination treatment of gefitinib and chloroquine slightly decreased the PC9/GR cell proliferation only at 24 h. However, the addition of YAP knock down to the combined treatment strongly restored the sensitivity of gefitinib in PC9/GR cells at both 6 and 24 h (Figure 4A).

Next, we checked the effect of YAP inhibitor verteporfin in EGFR‐TKI‐resistant cells. Verteporfin is an FDA‐approved drug for treating macular degeneration and it is the most commonly used YAP inhibitor.^22^ Verteporfin can act as a photosensitizer and it also constrains YAP by disturbing the binding of YAP and its partner TEAD.^35^ Intriguingly, verteporfin was revealed to have a function as an autophagic blocker by causing oligomerization of p62.^36^, ^37^ Therefore, verteporfin is an ideal drug to explore the effect of inhibiting YAP‐p62 signaling on cell viability in EGFR‐TKI‐resistant cells. Verteporfin significantly decreased the expression of total p62, YAP as predicted, it also induced the oligomerization of p62 in part (Figure 4B,C; Figure S4A,B). Notably, verteporfin remarkably decreased the expression of PD‐L1 which is a target for cancer immunotherapy and one of the transcriptional targets of YAP (Figure 4B). To check the combinational effect of verteporfin with autophagy blocker or EGFR‐TKI in PC9/GR, we treated verteporfin with chloroquine or gefitinib. Congruous to the previous results, gefitinib treatment decreased the expression of p62 and, combination treatment of chloroquine and gefitinib caused the accumulation of p62 (Figure 4D; Figure S4C). But notably, the combination of verteporfin and gefitinib with or without chloroquine markedly decreased the expression p62, PD‐L1, and YAP at the same time (Figure 4D). Combination treatment of verteporfin and gefitinib markedly induced the cleavage of PARP (Figure 4D). Consistently, CCK assay revealed that although the combination of chloroquine and gefitinib was not effective for decreasing cell viability after 6 h treatment, the combined treatment of gefitinib with verteporfin significantly diminished the cell viability of PC9/GR cells regardless of chloroquine (Figure 4E). To validate the effect of verteporfin on inhibiting PC9/GR cells, we treated several different doses of verteporfin. In single treatment with verteporfin, high dose of verteporfin was more effective to suppressing the proliferation of PC9/GR cells. However, in the combination of verteporfin with gefitinib or chloroquine, there were no significant differences between high dose and low dose of verteporfin (Figure S4D). These findings indicate that verteporfin has a promising pharmacological effect in inhibiting cell proliferation of EGFR‐TKI‐resistant cell with decreasing YAP, p62, and PD‐L1 simultaneously.

### 3.5 The expressions of YAP and p62 have a positive correlation in EGFR‐mutant human lung adenocarcinoma

**FIGURE 5:**
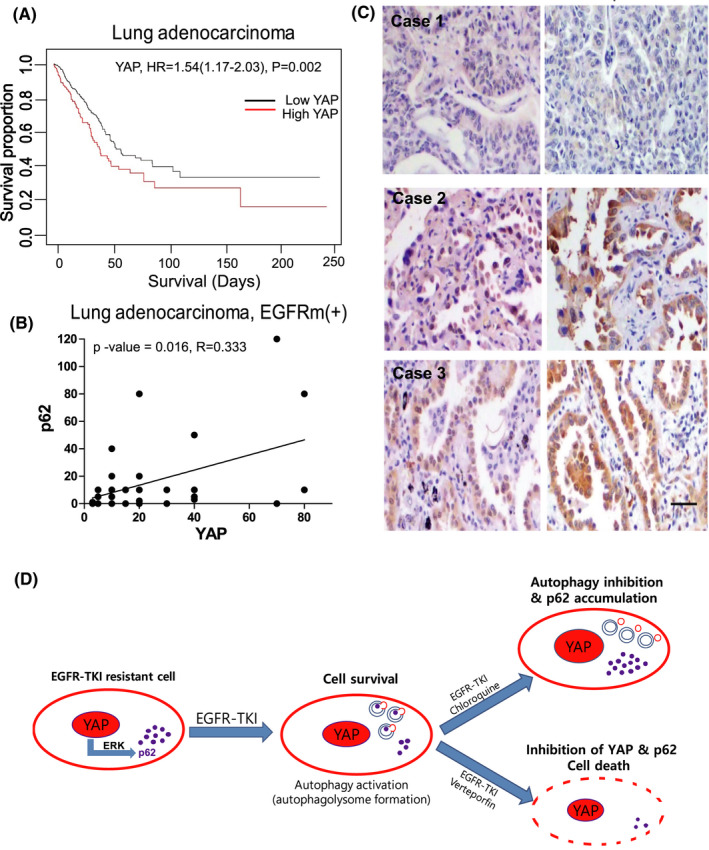
YAP has a positive correlation with p62 in EGFR‐mutant lung adenocarcinoma. (A) Survival analysis of YAP mRNA expression in lung adenocarcinoma according to the TCGA data (*n* = 576). (B) Correlation analysis of p62 and YAP of EGFR mutated lung adenocarcinoma patients of Chungnam national university hospital by IHC (*n* = 27). (C) These are three representative pictures of IHC staining for p62 and YAP in lung adenocarcinoma patients. #1 patient had both low expressions of YAP and p62, while #2 and #3 patients had high expressions of both. (D) Graphical summary of YAP and p62 relationship in EGFR‐TKI‐resistant lung adenocarcinoma. YAP regulates the transcription of p62 via ERK in EGFR‐TKI‐resistant cell. Autophagy blocker chloroquine induces the significant accumulation of oncogenic p62. YAP inhibitor verteporfin significant decreases the expression of YAP and p62

Lastly, we tried to identify the clinical implication of YAP and p62 in lung cancer. Based on the log‐rank test and TCGA database, high mRNA expression of *YAP1* was also significantly associated with poor overall survival in patients with lung adenocarcinoma [*n* =576, HR, 1.54; 95% CI: 1.17–2.03; *p*‐value = 0.002] (Figure 5A). These data suggest that YAP is positively correlated with poor prognosis of lung adenocarcinoma patients (Figure 5B). Next, we examined the expressions of YAP and p62 in lung adenocarcinoma patients who have EGFR mutations using Chungnam national university hospital patients’ samples. Consistently, the results also demonstrated that YAP expression has a positive correlation with p62 expression (*p*‐value = 0.016, *R* = 0.333) (Figure 5B,C). Patients with high YAP expression tended to have high p62 expression, supporting our previous experimental conclusion that YAP regulates p62 expression in EGFR‐mutant lung adenocarcinoma.

## 4 DISCUSSION

The resistance to various chemotherapeutic drugs, including target therapies and immune checkpoint inhibitors, is one of the most serious difficulties in cancer treatment. Third generation EGFR‐TKIs such as Osimertinib has potent effect for EGFR T790M‐mutant lung cancer, however there are many other resistant mechanisms which have no specific therapeutic target therapy. To overcome drug resistance, new attempts have been made to target cell metabolism, microbial populations, and autophagy.^4^, ^14^, ^29^ Since autophagy not only has physiological functions but also participates in anticancer resistance, many studies targeting autophagy have been conducted.^11^, ^13^, ^30^ Among autophagy inhibitors, chloroquine is the most commonly used, and it was originally FDA‐approved for the treatment of malaria. Chloroquine blocks the autophagic flux by preventing the fusion of autophagosome and lysosome.^27^ Like autophagy, which has a variety of roles in cancer, autophagy inhibitors can play different functions depending on contexts. So far, the effect of autophagy inhibitor has been controversial, and deciding the appropriate indication for autophagy inhibitors in cancer is a very important point of clinical study.

In this study, we confirmed that both EGFR‐TKI‐sensitive and ‐resistant cells activate autophagy after EGFR‐TKI treatment. Although PC9/GR cells already have acquired EGFR‐TKI resistance after long exposure of EGFR‐TKI, they also activate autophagy in response to EGFR‐TKI treatment. This suggests that both cells use autophagy as one of the survival mechanisms to EGFR‐TKI. Many studies also demonstrated that chemotherapy activates autophagic flux in several cancer cells and in some studies, autophagic inhibitors are effective when combined with those chemotherapeutic drugs.^24^, ^38^

However, our results showed that autophagic inhibitor, chloroquine causes a significant accumulation of oncogenic p62 which promotes cell proliferation and invasion in PC9 and PC9/GR cells. This inevitable accumulation of p62 might decrease the effect of chloroquine in EGFR‐TKI‐resistant cells. Although oncogenic roles of p62 have been studied in many cancer,^13^, ^39^, ^40^ there are few studies that deeply investigated the effect of accumulated p62 caused by chloroquine.^24^ We confirmed that the Hippo effector YAP regulates the transcription of p62 via ERK signaling for the first time. Our study also showed that when siYAP was treated, the accumulation of p62 by autophagy inhibitor was significantly suppressed. We revealed that verteporfin is helpful to overcome the accumulation of p62 accompanied by autophagy inhibitor and the resistance of EGFR‐TKI in lung cancer.

In this study, we used verteporfin which has variable targets including YAP, p62, and autophagy.^36^, ^41^ Verteporfin was approved by FDA and it is safely used for photodynamic therapy. A more recent study showed verteporfin induces lysosomal membrane permeabilization and improves the anticancer effect of sorafenib in hepatocellular carcinoma.^42^ Interestingly, we found that verteporfin also effectively decreased the expression of immunotherapy target, PD‐L1. YAP is a well‐recognized poor prognostic factor in lung cancer patients.^15^ And, many clinical studies also showed that high expression of p62 was significantly associated with aggressive tumor characteristics^43^ and overexpression of p62 is correlated with poor overall survival in many cancer patients. We also confirmed the overexpression of YAP has a positive correlation with poor prognosis of lung adenocarcinoma, respectively. Our clinical results showed for the first time a noteworthy positive correlation between YAP and p62 in EGFR‐mutant lung cancer patients’ tissues.

Generally, p62 is known to be regulated by multiple signals including ERK in several contexts.^11^, ^12^ And, ERK is known as one of the down‐streams of YAP and our previous data also demonstrated YAP activates ERK signaling in lung cancer cell lines including A549 and PC9.^17^ In addition, Haibin et al. showed that YAP promotes gastric cancer cell survival/migration/invasion via ERK/endoplasmic reticulum stress pathway.^44^ In lung cancer with EGFR mutations, ERK signaling is expected to be constitutively activated. But our experimental data indicated that even though ERK signaling is activated in EGFR‐mutant lung adenocarcinoma such as PC9 cells, overexpression of YAP can induce ERK signaling even more (Figure 3B). Conversely, knock down of YAP significantly decreased the phosphorylation of ERK in PC9/GR cells (Figure 3C). We believe that basal expression of ERK signaling is upregulated by mutant EGFR in lung cancer patients, but the addition of the hyperactive YAP can increase the ERK activity, and then upregulate the expression of p62. Although there are complicated regulatory mechanisms on p62, we suggest that YAP‐ERK signaling can control the expression of p62 in lung cancer and our clinical data support the clinical relevance of interaction between YAP and p62 in lung adenocarcinoma. These results also suggest future clinical investigation with combinational targeting YAP and p62 might be very meaningful.

In conclusion, p62 that is accumulated by autophagy inhibitor chloroquine has oncogenic functions which induce cell survival and invasion in EGFR‐TKI‐resistant cells. So, although autophagy is a novel target for overcoming EGFR‐TKI resistance, the effect of oncogenic p62 should be carefully considered when using autophagy inhibitors, especially drugs that act at the end of autophagy, such as chloroquine and bafilomycin A1. In addition, we confirmed that YAP regulates the transcription of p62 via ERK, and YAP inhibition can significantly decrease the expression of p62 in EGFR‐TKI‐resistant lung cancer. Our findings demonstrate that targeting YAP‐p62 will be helpful to suppress the EGFR‐TKI resistance of lung adenocarcinoma (Figure 5D). And, drug repurposing of verteporfin for lung cancer treatment is valuable to consider because it can inhibit critical targets: p62, YAP, and PD‐L1 at the same time.

## CONFLICT OF INTEREST

The authors have no conflict of interest.

## AUTHOR CONTRIBUTIONS

Ju‐Ock Kim, Hee Sun Park, Jeong Eun Lee, Chaeuk Chung, and Eun‐Kyeong Jo provided scientific ideas and designed the study. Cheaeuk Chung drafted the manuscript and designed the figures. Geon Yoo and Da‐Hye Lee edited and modified the manuscript. Dahye Lee performed the cell experiments. Eunyoung Moon, Yang Hoon Huh, and Sang‐Hee Lee performed the electron microscopy experiments and analyzed the data. Min‐Kyung Yeo and Goeun Bae performed and analyzed the experiments with human specimens. Da Hyun Kang and Sang Yeon Cho performed the TCGA data analysis.

## ETHICS APPROVAL STATEMENT

Written informed consent was obtained from all patients and the study protocol was approved by the Clinical Research Ethics Committee of Chungnam national university hospital. Institutional review board (IRB) approved this research. IRB file number is 2015‐07‐001‐002. All experiments were performed in accordance with relevant guidelines and regulations.

## Supporting information

Fig S1

Fig S2

Fig S3

Fig S4

Fig S5

Supplementary Material
