# Supplementary material for: Targeting YAP‐p62 signaling axis suppresses the EGFR‐TKI‐resistant lung adenocarcinoma
Source: Cancer Med. 2021 Jan 23;10(4):1405–17. doi: 10.1002/cam4.3734 (PMC7926029; doi:10.1002/cam4.3734)
Supplement: Supplementary file 1 — Fig S1 [file CAM4-10-1405-s001.docx]

**Fig. S1**

**Fig. S1**. (A) Original immunoblots of YAP, p62, LC3-I, LC3-II, and β-actin in **Fig. 1A**. Red rectangle indicates the cropped representative image in **Fig. 1**
