# Supplementary material for: Targeting YAP‐p62 signaling axis suppresses the EGFR‐TKI‐resistant lung adenocarcinoma
Source: Cancer Med. 2021 Jan 23;10(4):1405–17. doi: 10.1002/cam4.3734 (PMC7926029; doi:10.1002/cam4.3734)
Supplement: Supplementary file 2 — Fig S2 [file CAM4-10-1405-s002.docx]

**Fig. S2**

**
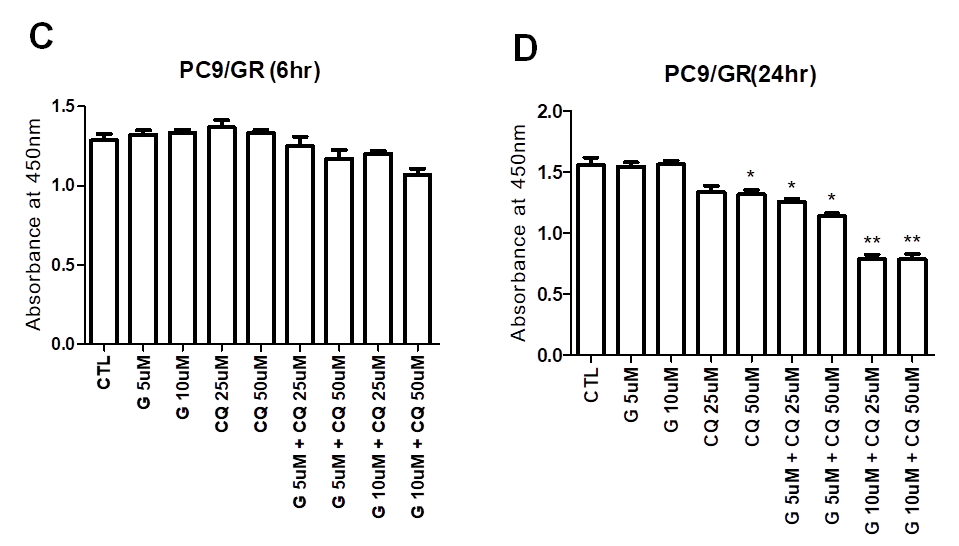
**

**
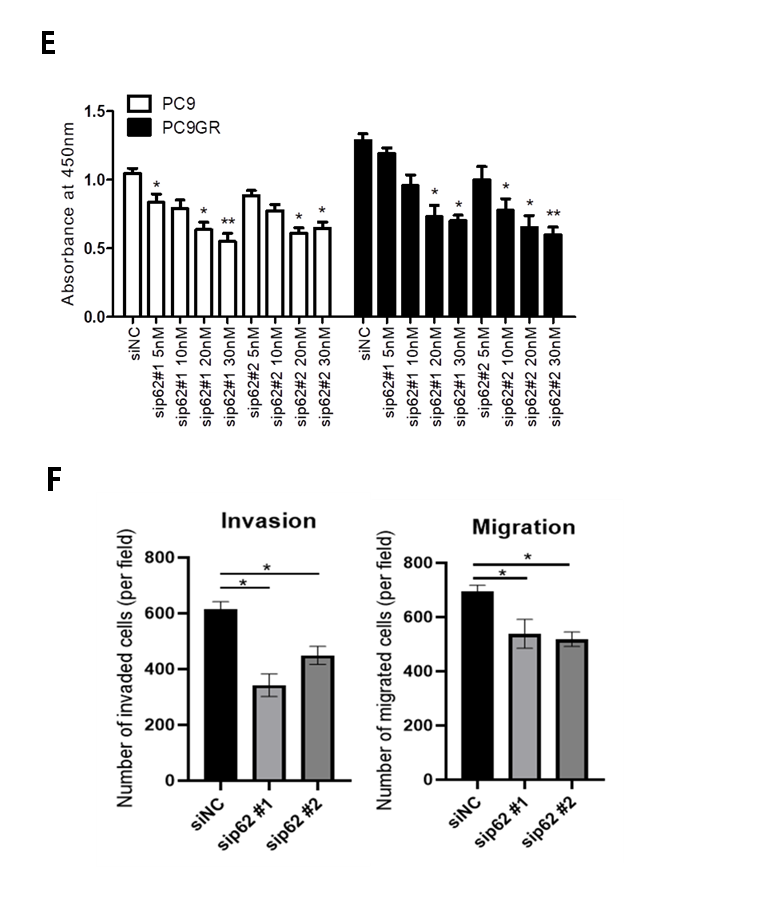
**

**Fig. S2**. (A) Original immunoblots of YAP, p62, LC3-I/II and β-actin in **Fig. 2A**. (B) Original immunoblots of YAP, p62, LC3-I/II, and β-actin in **Fig. 2D**. Red rectangle indicates the cropped representative image in **Fig. 2.** (C and D) The CCK assay of PC9/GR cells after treatment of different doses of gefitinib or chloroquine at 6hr and 24hr, respectively. (E) The CCK assay of PC9 and PC9/GR cells after treatment of multiple doses (5, 10, 20, and 30uM) of p62 siRNA. (F) The quantification of the results of the migration and invasion assay. We used a non-coated transwell membrane for cell migration assay and transwell membrane coated with 2mg/ml Matrigel was used for cell invasion assay. The HCC827/GR cell lines were transfected with the vector or p62 siRNA.
