# Supplementary material for: Targeting YAP‐p62 signaling axis suppresses the EGFR‐TKI‐resistant lung adenocarcinoma
Source: Cancer Med. 2021 Jan 23;10(4):1405–17. doi: 10.1002/cam4.3734 (PMC7926029; doi:10.1002/cam4.3734)
Supplement: Supplementary file 3 — Fig S3 [file CAM4-10-1405-s003.docx]

**Fig. S3**

**
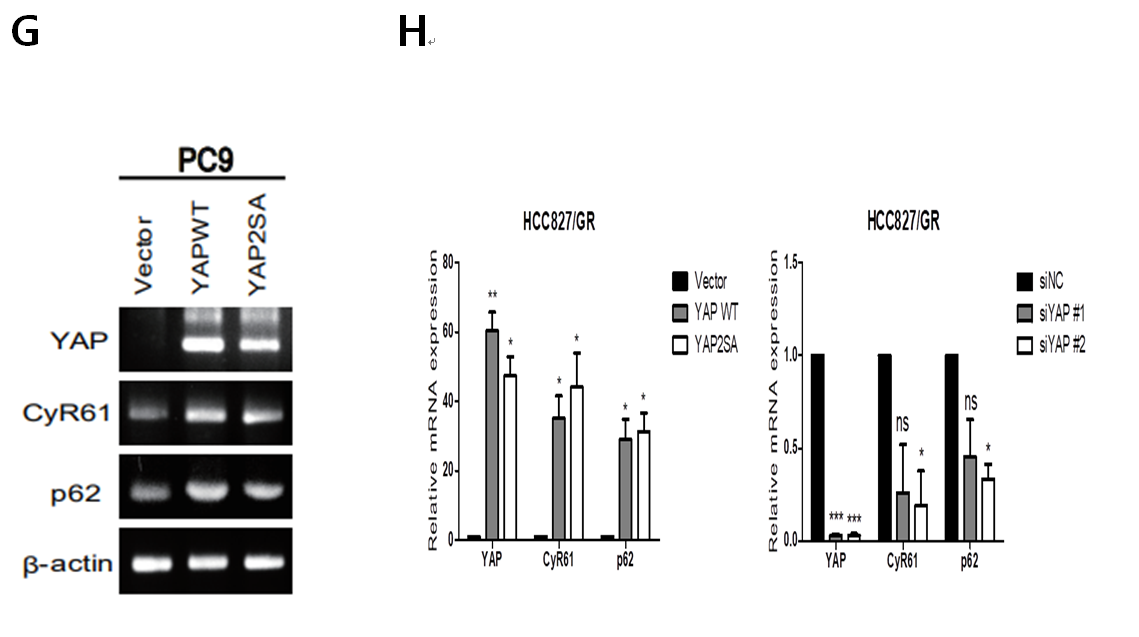
**


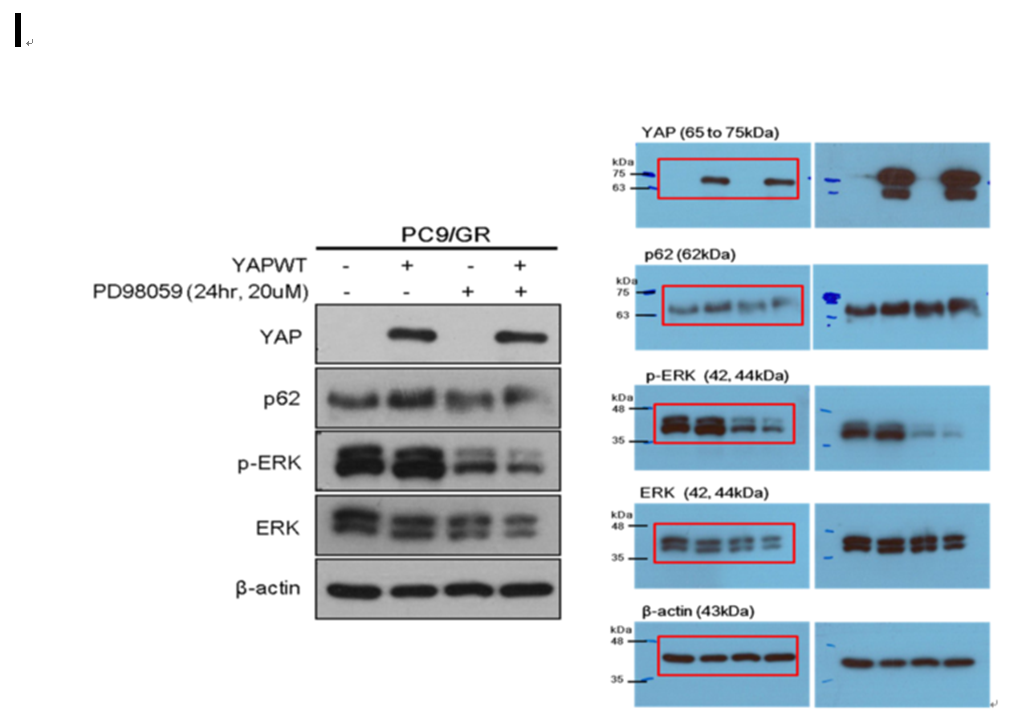


**Fig. S3**. (A) Original immunoblots of YAP, p62, LC3-I/II, and β-actin in **Fig. 3A**. (B) Original immunoblots of YAP, p62, LC3-I/II, p-ERK, ERK, and β-actin in **Fig. 3B.** (C) Original immunoblots of YAP, p62, LC3-I/II, p-ERK, ERK, and β-actin in **Fig. 3C**. (D) Original immunoblots of YAP, p62, LC3-I/II, PARP, C-PARP and β-actin in Fig. **3D**. (E) Original immunoblots of YAP, p62, LC3-I/II, and β-actin in Fig. 3E. (F) Original immunoblots of p-ERK, ERK, p62 and β-actin in Fig. 3F. Red rectangle indicates the cropped representative image in **Fig. 3.** (G) Right panel shows the mRNA expressions of YAP, Cyr61, p62, and β-action determined by RT-PCR. (H) The left panel shows qRT-PCR data of p62 in HCC827/GR cell after overexpression of YAP WT or YAP 2SA. The right panel shows qRT-PCR data of p62 in HCC827/GR cell after knock-down of YAP by using YAP siRNA #1 and #2. (I) PC9/GR cells was over-expressed with WT YAP and treated with control or ERK inhibitor, PD98059. Left panel is the western blot of PC9/GR of YAP, p62, p-ERK, ERK and β-actin. Right panel is the original immunoblots.
