# Supplementary material for: Targeting YAP‐p62 signaling axis suppresses the EGFR‐TKI‐resistant lung adenocarcinoma
Source: Cancer Med. 2021 Jan 23;10(4):1405–17. doi: 10.1002/cam4.3734 (PMC7926029; doi:10.1002/cam4.3734)
Supplement: Supplementary file 4 — Fig S4 [file CAM4-10-1405-s004.docx]

**Fig. S4**

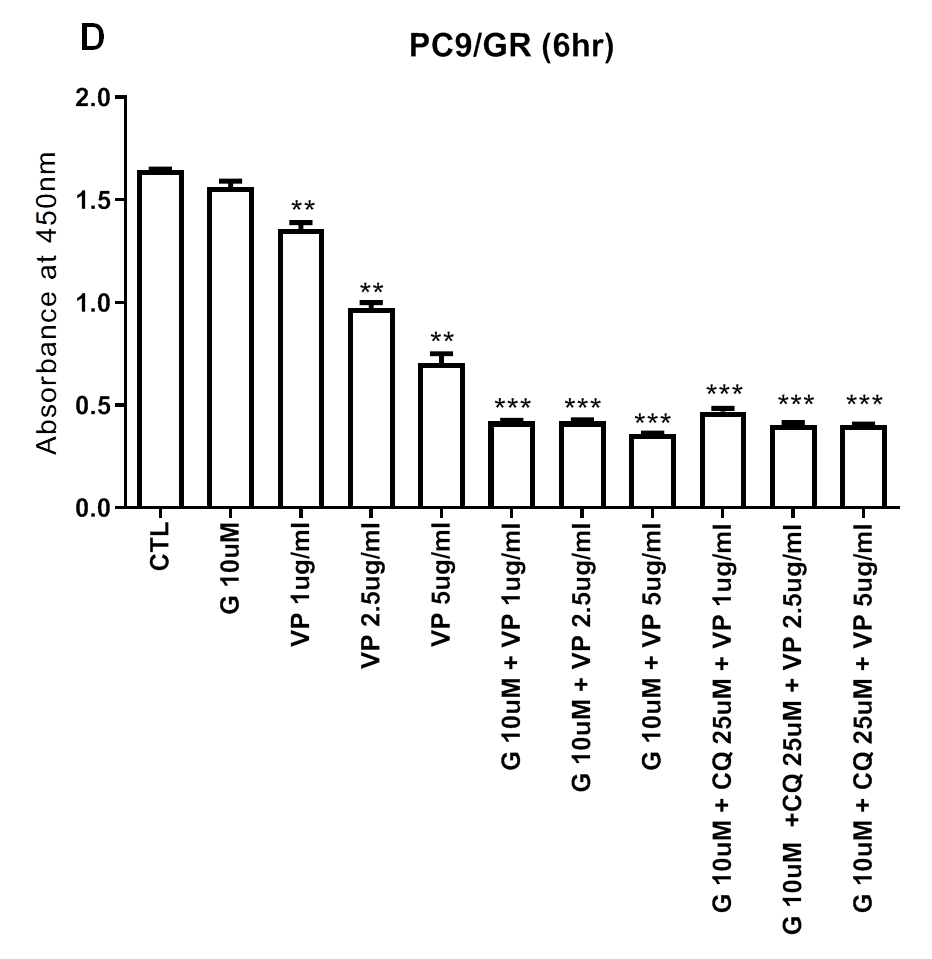


**Fig. S4**. (A) Original immunoblots of YAP, PD-L1, p62 and β-actin in **Fig. 4B**. (B) Original immunoblots of YAP, p62 and β-actin in **Fig. 4C.** (C) Original immunoblots of YAP, PD-L1, p62, C-PARP, PARP and β-actin in **Fig. 4D.** Red rectangle indicates the cropped representative image in **Fig. 4.** (D) The CCK assay of PC9/GR cells after several different doses of verteporfin treatment with gefitinib or chloroquine.
