# Supplementary material for: Targeting YAP‐p62 signaling axis suppresses the EGFR‐TKI‐resistant lung adenocarcinoma
Source: Cancer Med. 2021 Jan 23;10(4):1405–17. doi: 10.1002/cam4.3734 (PMC7926029; doi:10.1002/cam4.3734)
Supplement: Supplementary file 5 — Fig S5 [file CAM4-10-1405-s005.docx]

Fig. S5.

(A)


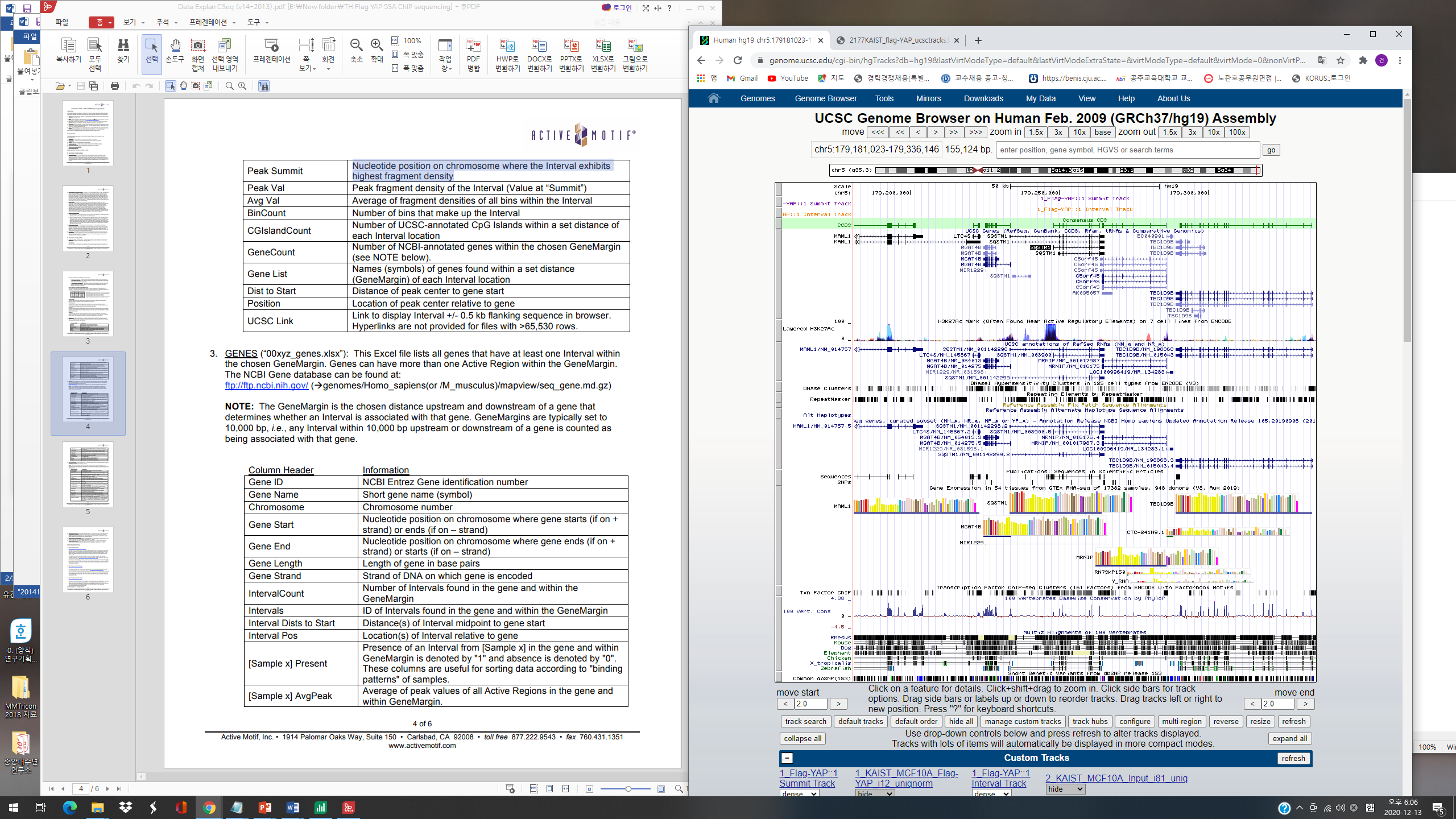


(B)


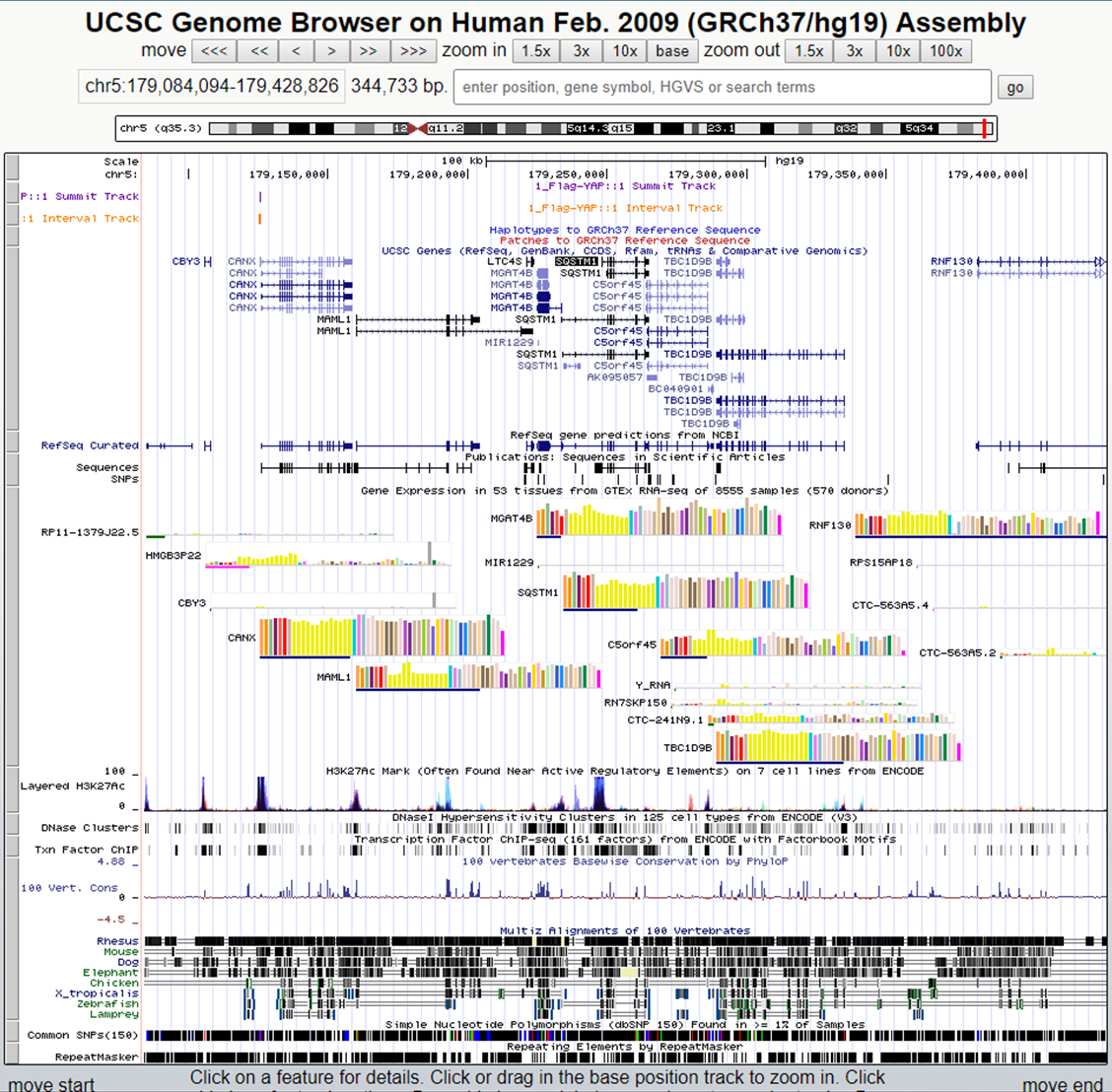
We added these contents in the result part and M&M part.

Figure 2. YAP ChIP sequencing result indicating YAP is enriched near CANX promoter region.

(C)


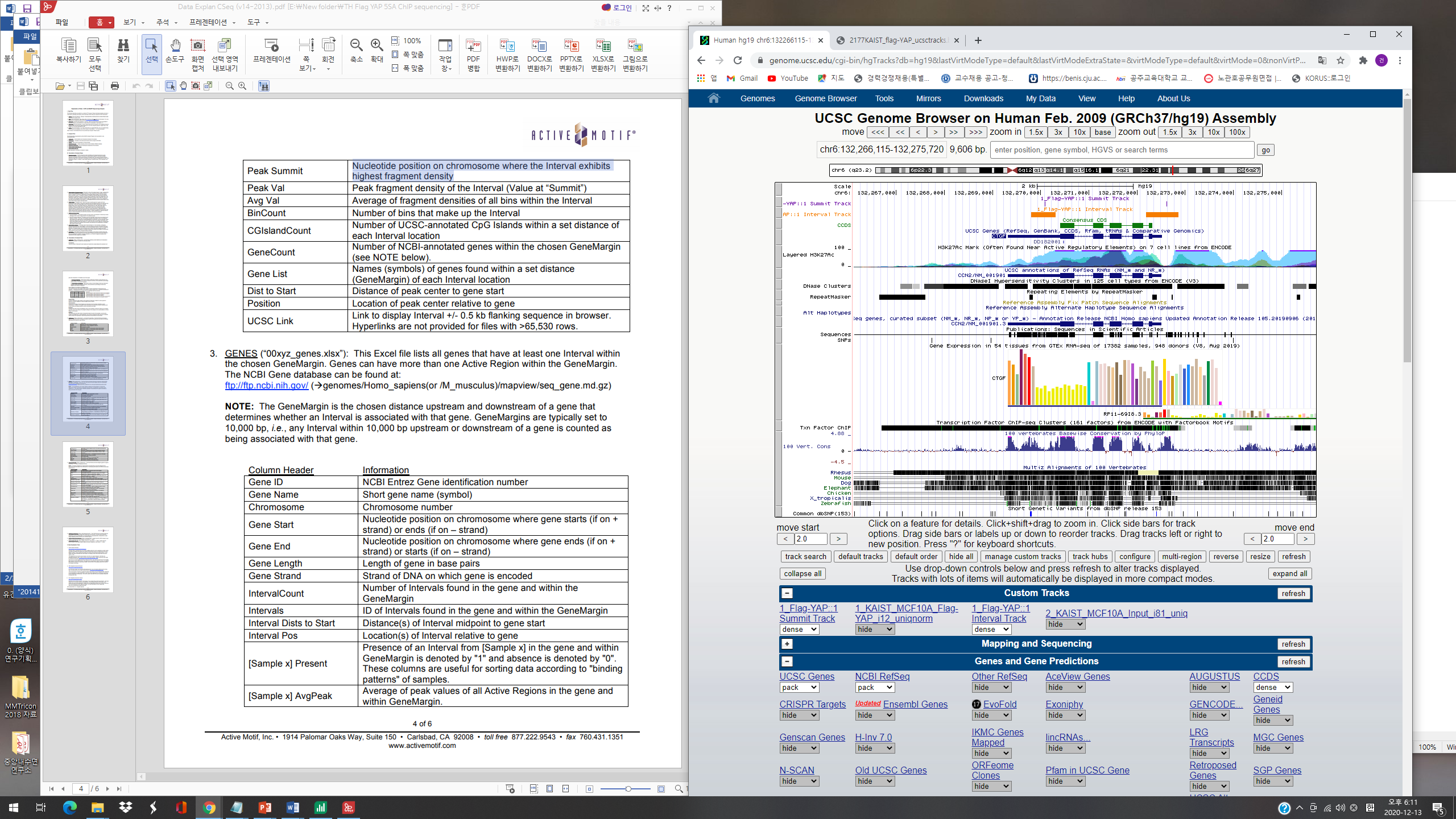


Figure 3. YAP ChIP sequencing result indicating YAP is enriched near chromatin region of its well-known transcription target, CTGF.

Fig. S5. ChIP sequencing analysis of YAP .

(A) YAP ChIP sequencing result indicating YAP is not enriched in region near SQSTM1 (gene encoding p62) near promoter or enhancer region. (B) YAP ChIP sequencing result indicating YAP is enriched near CANX promoter region. (C) YAP ChIP sequencing result indicating YAP is enriched near chromatin region of its well-known transcription target, CTGF.

We had searched for possible YAP binding region within the p62 promoter or enhancer using Flag-YAP ChIP seq data in MCF-10A (unpublished) from Dr. Dae-Sik Lim (KAIST). However, we could not find nucleotide position near promoter or potential enhancer region with the significant peak density. Therefore, we could not conclude that YAP directly binds to p62 chromatin region. Raw and processed ChIP sequencing data from overexpressed YAP enrichment in the genome was unpublished data by Dr. Dae-Sik Lim (KAIST). ChIP sequencing data were uploaded onto UCSC genome browser (<http://genome.ucsc.edu>).
