# Supplementary material for: Targeting YAP‐p62 signaling axis suppresses the EGFR‐TKI‐resistant lung adenocarcinoma
Source: Cancer Med. 2021 Jan 23;10(4):1405–17. doi: 10.1002/cam4.3734 (PMC7926029; doi:10.1002/cam4.3734)
Supplement: Supplementary file 6 — Supplementary Material [file CAM4-10-1405-s006.docx]

**Supplementary Materials & Methods**

**Western blotting assay**

We harvested and suspended cells in protein lysis buffer (Translab). For subcellular fractionation, the protein was extracted using NE-PER Nuclear and Cytoplasmic Extraction reagents (Thermo Fisher Scientific). Protein concentration was determined by means of the Bio-Rad protein assay (Bio-Rad, cat.no.500-0006). We separate 30 uL of cell lysate on a 10% SDS-PAGE gel and then transferred to polyvinylidene difluoride (PVDF) membrane (Millipore). We used the following antibodies: anti-β-actin (sc-47778, Santa Cruz Biotechnology), anti-YAP (#4912S, Cell signaling), anti-PD-L1 (#13684, Cell signaling), anti-p62 (P0067, Sigma), anti-LC3 (L8918, Sigma), and anti-PARP (#9542, Cell signaling), anti-p-ERK (#9101, Cell signaling), anti-ERK (#9102, Cell signaling).We developed blots by using an enhanced chemiluminescence detection kit (Thermo).

**RT-PCR**

We collected cells for RNA extraction. Total RNA was isolated using TRIzol reagent (Invitrogen),as said by the instructions of manufacturer.cDNA was synthesized using Maxime RT premix (Oligo(dT) primers, Intron). PCR was performed using Ex Taq DNA polymerase (Takara). All experiments were performed in triplicate. The primers used for PCR amplification were as follows: (a) YAP mRNA (sense 5'-GAA CCA GAG AAT CAG TCA GA-3', and antisense 5'-GGA TTG ATA TTC CGC ATT GC-3'), (b) CyR61 mRNA (sense 5'-CCT TGT GGA CAG CCA GTG TA-3', and antisense 5'-ACT TGG GCC GGT ATT TCT TC-3') (c) p62 mRNA (sense 5'-TGT GTA GCG TCT GCG AGG GAA A-3', and antisense 5'-AGT GTC CGT GTT TCA CCT TCC G-3'), (d) β-actin mRNA (sense 5'-AGG CCC AGA GCA AGA GAG G-3', and antisense 5'-TAC ATG GCT GGG GTG TTG AA-3'). We electrophoresed PCR products on a 1% agarose gel and then visualized them byusing ethidium bromide staining.

**SiRNA-mediated gene expression knockdown**

Small interfering RNA (siRNA) directed against YAP#1 (sense 5’-CUG GUC AGA GAU ACU UCU UAA TT-3’, antisense 5’- UUA AGA AGU AUC UCU GAC CAG TT-3’), YAP#2 (sense 5’-GCC ACC AAG CUA GAU AAA GAT T-3’, antisense 5’-UCU UUA YCU AGC UUG GUG GCT T-3’), p62#1 (sense 5’-GAG GAU CCG AGU GUG AAU UUC CUC TT-3’, antisense 5’-GAG GAA AUU CAC ACU CGG AUC CUC TT-3’), p62#2 (sense 5’-GAC ACC AUC CAG UAU UCA AAG CUC TT-3’, antisense 5’-GAG CUU UGA AUA CUG GAU GGU GUC TT-3’), and Negative control (sense 5’-UUC UCC GAA CGU GUC ACG UTT-3’, antisense 5’-ACG UGA CAC GUU CGG AGA ATT-3’) were synthesized by Gene Pharma. We introduced 10 nM (YAP) siRNA into cells by transient transfection with RNAi MAX (Invitrogen) in accordance as said by the instructions of manufacturer.

**Immunohistochemical staining analysis**

We mounted tissue sections on the coated slides, deparaffinized with xylene, hydrated in serial solutions of alcohol. Then we heated the slides in a pressure cooker containing 10 mmol/L sodium citrate (pH 6.0) for 3 minutes at strong power for antigen retrieval. We performed endogenous peroxidase activity blocking by using 0.03% hydrogen peroxide containing sodium azide for 5 minutes. We incubated the sections at room temperature for 4 h with anti-YAP (#4912S, Cell signaling) and anti-p62 (P0067, Sigma). After washing, the samples were incubated in labeled polymer-HRP anti-mouse (DakoEnVision+system-HRP (DAB), Dako, Carpinteria, California, USA) for an additional 20 minutes at room temperature followed by additional washing. After rinsing, chromogen was developed for 2 minutes. The slides were then counterstained with Meyer's hematoxylin, dehydrated, and then covered by a coverslip. We scored immunohistochemical staining to evaluate both intensities of immunohistochemical staining and the proportion of stained tumor cells in each stained slide. We scored the intensity as 0 (negative), +1 (mild), +2 (moderate), +3 (marked) and proportions were scored ranged from 0 to 100%.

**Transmission electron microscopy**

We sequentially fixed samples from PC9 and PC9/GR cells with 2.5% glutaraldehyde and 1% osmium tetroxide on ice for 2 h and washed with PBS. We dehydrated the tissues in ethanol and propylene oxide series, embedded in Epon 812 mixture, and polymerized in an oven at 70°C for 24 h. The sections acquired from polymerized blocks were collected on 150 mesh copper grids, counterstained with uranyl acetate and lead citrate, and examined with Bio-HVEM system (JEM-1400Plus at 120 kV and JEM-1000BEF at 1000 kV, JEOL, JAPAN).
